# Sporadic renal hemangioblastoma: A case report of a rare benign renal tumor

**DOI:** 10.1002/ccr3.2466

**Published:** 2019-10-07

**Authors:** Lukas Oberhammer, Michael Josef Mitterberger, Lukas Lusuardi, Thomas Kunit, Martin Drerup, Daniela Colleselli, Hubert Griessner, Martina Hager

**Affiliations:** ^1^ Department of Urology and Andrology Uniklinikum Salzburg Salzburg Austria; ^2^ Department of Pathology Uniklinikum Salzburg Salzburg Austria

**Keywords:** carcinoma, case report, hemangioblastoma, kidney, renal cell, von Hippel‐Lindau disease

## Abstract

In renal tumors, suspicious for renal cell carcinoma, where there is any doubt and discrepancy between morphology and immune profile, we recommend performing further immunohistochemical staining for pan‐cytokeratin, S100, NSE, and inhibin‐alpha. Thus, follow‐up overtreatment can be avoided in cases of benign kidney tumors.

## Graphical abstract

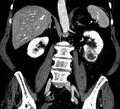

## 1 BACKGROUND

Hemangioblastoma is a benign tumor of mesenchymal cell proliferation and normally occurs in the central nervous system (CNS), mainly in the cerebellum. Most of these tumors emerge sporadically, while approximately 20%‐25% are associated with the von Hippel‐Lindau (VHL) disease.^1^, ^2^ VHL disease is a rare genetic disorder with mutations of the VHL tumor suppressor gene and can cause various benign and malignant tumors, particularly in the CNS but also in the internal organs.^3^

In some cases, hemangioblastomas are located externally usually combined with VHL disease. The VHL gene is located on chromosome 3p25 and encodes for a tumor suppressor protein. Mutations result in a loss of function of the protein complex, which causes an accumulation of hypoxia‐inducible factors. As a result, the transcription of hypoxia‐responsive genes involving cell proliferation, angiogenesis, erythropoiesis, and other proangiogenetic factors are initiated. These factors often cause the development of vascular tumors**.** 4

However, sporadic renal hemangioblastoma (RH) without VHL disease is very rare. The morphological character is more less the same as hemangioblastomas occurring in the CNS as they show both oval and polygonal cells with pale or eosinophilic cytoplasm. Typically, prominent vascularity with thin‐walled and thick‐walled blood vessels is seen. Hemangioblastomas can be easily misdiagnosed for a renal cell carcinoma because of similar histological and immunohistochemical features.^5^

So far, only 14 cases of RH have been reported. Compared to the World Health Organization (WHO) World Cancer Report 2014, kidney cancer is the ninth most common cancer in men and 14th most common cancer in woman with approximately 330 000 cases in 2012.^6^, ^7^ The differences in adjuvant treatment and prognosis make it important to be able to differentiate between the rarely occurring RH and the much more frequent renal cell carcinoma.

We present one of (very) few cases of an isolated RH, admitted to our Department of Urology, identifying its pathological features and discussing a review of the literature.

## 2 CASE PRESENTATION

**Figure 1:**
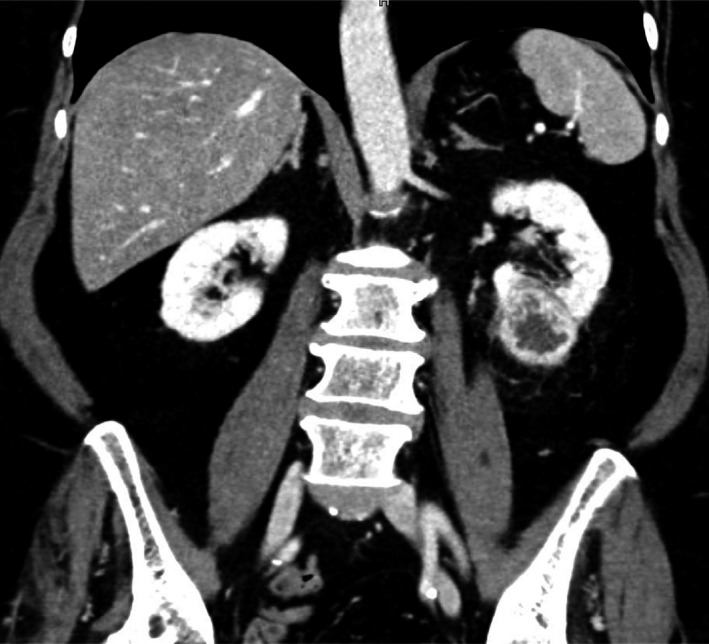
CT scan, preoperative. A 4.2 × 3.6 × 4.3 cm large mass in the lower pole of the left kidney

In April 2016, a 72‐year‐old woman, with a renal mass in her left kidney was admitted to our department for further examination. An abdominal computed tomography (CT) scan showed a 4.2 × 3.6 × 4.3 cm large tumor in the lower pole of the kidney, with 20‐160 Hounsfield units with heterogeneous contrast enhancement, except for a hypodense region in the center (Figure 1). These findings aroused the suspicion of a renal cell carcinoma. A chest CT scan showed no evidence of metastasis.

The patient was asymptomatic and did not show urinary symptoms, such as microhematuria or abdominal pain. Laboratory examination revealed normal findings. The patient's family history included two sisters with breast cancer and a father with lung cancer. Otherwise, there was no history of neoplastic disease for over four generations. In addition, renal scintigraphy was performed and revealed a general loss of renal function, with a calculated clearance of 75 mL/min, but no evidence of mechanical obstruction of the upper urinary tract.

**Figure 2:**
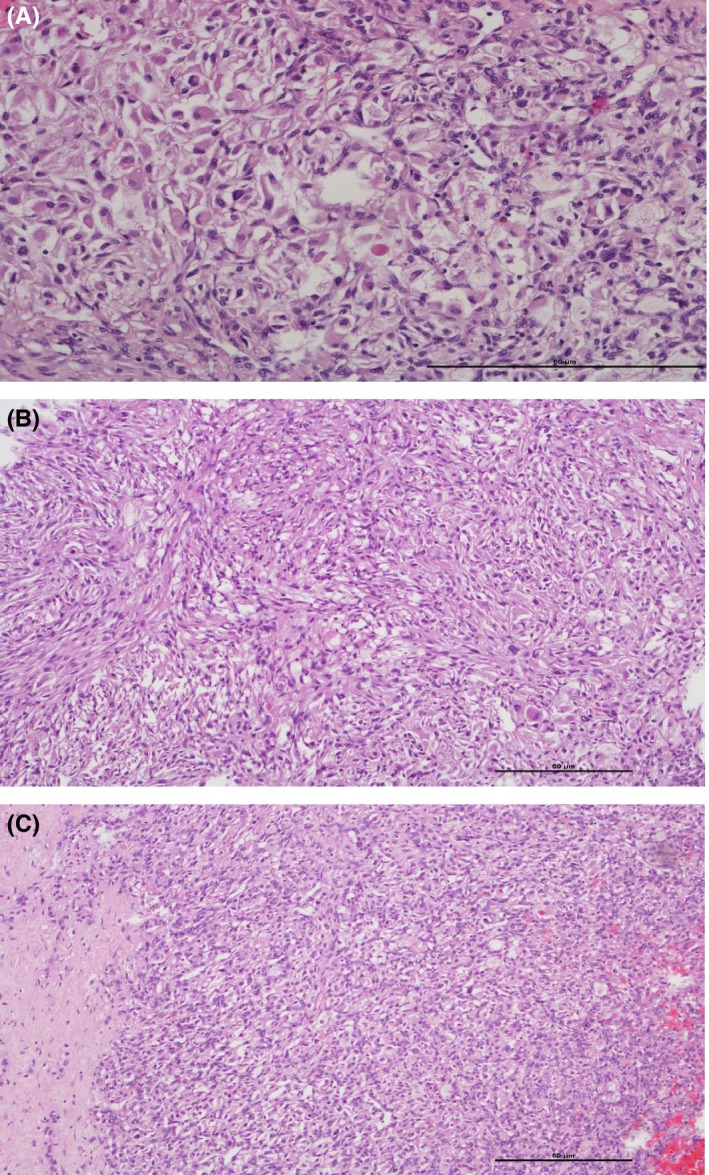
Histological examination demonstrated a renal hemangioblastoma with (A) rhabdoid morphology and clear cytoplasma, H&E stain, 200×. In addition the tumour showed (B) areas of spindle cells, H&E stain, 100×, as well as (C) a prominent vascularity and sharp demarcation, H&E stain, 100×

Later on, the patient underwent a laparoscopic partial nephrectomy under general anesthesia. In warm ischemia, the tumor was excised within 25 minutes. After its removal, the specimen was put in a box with a mixture of water and formaldehyde and was send to our department for pathology. Next, gross examination and histology were performed (Figure 2). Paraffin blocks, H&E sections and further stainings for immunohistochemistry were sliced and pathological findings led us finally to diagnose a hemangioblastoma.

The hospital stay of the patient was uncomplicated and her laboratory findings were stable. After a good recovery, the patient was discharged on the fifth postoperative day. A follow‐up CT scan 6 months after partial nephrectomy revealed no evidence of recurrence.

In addition, a genetic examination for VHL disease was performed. It revealed normal findings for the DNA sequencing of the VHL‐coding exons and normal findings on the multiplex ligation‐dependent probe amplification analysis of the VHL gene. Therefore, there was no evidence of VHL disease.

### 2.1 Pathological findings

The histological findings showed a well‐demarcated tumor with polygonal and spindle cells with occasionally enlarged and bizarre nuclei. The cytoplasm was pale partly clear and in some regions, eosinophilic. Some tumor cells showed small eosinophilic globules and vacuoles in their cytoplasm. Rhabdoid features, necrosis, calcification, and hemorrhagic areas were also seen. The proliferation was accompanied by prominent vascularity. The proliferation rate of the tumor, as measured with Ki‐67, was low (5%). No mitotic activity was observed. In immunohistochemical findings, the tumor cells diffusely expressed S100, inhibin‐alpha, neuron‐specific‐enolase (NSE), CD10, and vimentin. Only focally isolated cells showed positivity with EMA and a weak but definite expression for PAX8 and WT1. SMA showed only focal staining in scattered tumor cells beside the expression of vessels, which was also demonstrated for CD34. Negative staining results were found for AE1/AE3, HMB‐45, melan A, myogenin, RCC, CK818, TFE, and desmin. Immunohistochemical data and general characteristics are summarized in Tables 1, 2, and 3.

**Table 1:** Immunohistochemical markers

Case report	CD10	S100	Vimentin	Inhibin	PAX8	WT1	Pan‐cytokeratin	NSE	EMA	Necrosis
*Present Case*	+	+	+	+	+	+	−	+	focal +	+
Wang et al	n.i.	+	n.i.	+	n.i.	n.i.	n.i.	+	n.i.	+
Kurado et al	−	+	+	+	+	n.i.	−	n.i.	n.i.	n.i.
Doyle et al	n.i.	+	n.i.	+	+	n.i.	−	+	n.i.	−
Nonaka et al	n.i.	+	+	+	n.i.	−	−	n.i.	−	−
Verine et al	−	+	+	+	n.i.	n.i.	−	+	focal +	−
Ip et al	n.i.	+	n.i.	+	n.i.	n.i.	−	+	n.i.	−
Wang et al	n.i.	+	n.i.	+	n.i.	n.i.	n.i.	+	−	n.i.
Yin et al	+	+	+	+	n.i.	n.i.	−	+	focal +	+
Liu et al	n.i.	+	n.i.	+	n.i.	n.i.	−	+	−	−
Jiang et al	+	+	n.i.	+	n.i.	n.i.	−	+	n.i.	−
Zhao et al	+	+	+	+	+	n.i.	focal +	+	focal +	−

Abbreviation: n.i., no indication.

**Table 2:** Immunohistochemical markers, present case

Immunohistochemical panel *Present Case*	
Positive	Negative
CD10 *(Mako)*	RCC *(Cell marque)*
S100 *(Ventana)*	Pan‐cytokeratin *(Mako)*
Vimentin *(Ventana)*	CD34 *(Cell marque)*
Aktin *(Cell marque)*	Pan Melanoma *(Bio care)*
PAX8 *(Cell marque)*	Myogenin *(Cell marque)*
Inhibin *(Cell marque)*	CK8/18 *(Cell marque)*
WT1 *(Cell marque)*	Melan A *(Ventana)*
EMA *(Ventana)*	HMB45 *(Ventana)*
	CK7 *(Ventana)*
	Desmin *(Ventana)*
	TFE3 *(Cell marque)*

**Table 3:** General characteristics

Case report	Age (y)/gender	Chief complaint	Size (cm)	VHL (yes/no)	Follow‐up (mo)/prognosis
Wang et al	61/male	Asymptomatic	6.5	no	24/no recurrence
Kurado et al	37/male	Asymptomatic	3.6	no	n.i.
Doyle et al	3 cases	Hematuria Fever and weight loss Asymptomatic	n.i. n.i. n.i.	n.i. n.i. n.i.	n.i. n.i. n.i.
Nonaka et al	71/female	Asymptomatic	6.8	no	108/no recurrence
Verine et al	64/male	Other disease	3.2	no	12/no recurrence
Ip et al	58/male 55/female	Hematuria Low back pain	5.5 3.5	no no	24/n.i. 48/n.i.
Wang et al	29/male	Other disease	2.7	no	20/no recurrence
Yin et al	61/male	Asymptomatic	5.3	no	12/n.i.
Liu et al	16/female	Hematuria	1.2	no	6/no recurrence
Jiang et al	57/female	Asymptomatic	3	no	6/no recurrence
Zhao et al	51/female	Right‐sided lumbar abdominal pain	5.5	no	12/no recurrence

Abbreviation: n.i., no indication.

## 3 DISCUSSION

The present case showed a tumor with polygonal and spindle cells with enlarged nuclei and an eosinophilic but also clear cytoplasm. Some tumor cells demonstrated eosinophilic globules and vacuolization. Other areas with rhabdoid features and necrosis were also observed. Prominent vascularity was identified. Immunohistochemistry showed positivity of the tumor cells for CD10, PAX8, and WT1, which is a master control gene that is essential for kidney development, suggesting a tumor of renal origin.^8^ After negative staining for pan‐cytokeratin and RCC, which is a monoclonal antibody against renal tubule antigen that is positive in the majority of clear cell and papillary renal cell carcinomas, renal cell carcinoma was excluded.^9^ Additional immunhistochemical analysis was carried out and strong expression for S100, NSE, and inhibin‐alpha were observed. The immunoprofile in conjunction with the morphology and low proliferation rate led us to exclude a renal cell carcinoma and to diagnose a RH, which is the 15th reported case to date.

Hemangioblastoma is a rare benign mesenchymal tumor. It normally occurs in the CNS and is characterized by neoplastic stromal cells with a prominent vascularity.^10^ Doyle et al reported the pathological findings of 22 cases of peripheral hemangioblastomas outside the CNS (spinal nerve roots, soft tissues, kidneys, intestines, peritoneum, and orbit). All the tumors showed spindle and microvacuolated cells with eosinophilic or clear cytoplasmic contents, and all of them were characterized by intense vascularization. On immunohistochemical examinations, a considerable number of these hemangioblastomas positively expressed inhibin‐alpha (95%), neuron‐specific enolase (79%), and S100 (65%). In addition, markers detected in a few cases included weak expression for PAX8 and focal expression for EMA, desmin, and SMA. CD 31 and CD34 were only identified in endothelium of the capillary network but not in tumor cells.^11^

To date, 14 cases of a sporadic RH have been reported.^2^, ^5^, ^11^, ^12^, ^13^, ^14^, ^15^, ^16^, ^17^, ^18^, ^19^ In terms of the immunohistochemical findings, significant markers indicating diagnosis of RH include S100, inhibin‐alpha, and NSE. In all the reported cases, S100 and inhibin‐alpha expression were examined, and positivity was seen in 100% of the cases. NSE staining was performed in 12 of 14 cases of RH, and all of these tumors expressed it (Table 1). Most authors examined pan‐cytokeratin for exclusion of the major differential diagnosis, RCC. Staining for CD10, which is a marker expressed in the proximal tubular cells of the kidney and in the majority of clear cell and papillary renal cell carcinomas, was performed in 6 cases of RH and showed positive results in 50% of the cases. Also, PAX8, which is a transcription marker expressed in the renal tubular epithelial cells and in most renal cell carcinomas, was examined in 5 cases and showed positive results in 60% of the cases.^20^ For central and most peripheral hemangioblastomas outside the kidney no CD10 or PAX8 has been reported.^21^, ^22^ This supports the hypothesis that depending on the site of origin, hemangioblastomas have the capacity to express variable lines of differentiation.^16^, ^18^ An overview is shown in Table 1.

Interestingly, Montironi et al discussed whether the previously reported RHs are true RHs, or a diffuse hemangioblastoma‐like change in a clear cell renal cell carcinoma. The group of Montironi reported two cases of renal cell carcinomas each with two tumor components. The first part of the tumor consisting of hemangioblastoma‐like features and the second with morphology of a renal cell carcinoma. They described an acquired expression of inhibin‐alpha and S100 in the hemangioblastoma‐like part but not in the carcinoma cells. PAX8, CD10, and RCC were demonstrated in both components of the tumor. They believed that the hemangioblastoma‐like pattern could have a favorable prognostic significance.^23^ In the present case, inhibin‐alpha and S100 expression was consistently positive throughout the tumor and differed not between various regions.

In addition, foci with necrosis and cell atypia were found in the present case. In previous reports, necrosis in RH was observed in 18% (2/11) of the cases (see Table 1). In different types of tumors, necrosis, in combination with cellular atypia, can be indicative of malignancy.^24^, ^25^ In addition, necrosis is associated with aggressive behavior in some types of malignant tumors, especially in RCCs.^26^ In this context, it is important not to misjudge the dignity of the present tumor based only on these morphological features.

## 4 CONCLUSION

Renal hemangioblastoma is a very rare benign tumor, and a diagnosis cannot be made with radiographic techniques alone, such as CT or magnetic resonance imaging. Surgical excision of the tumor is needed to perform a precise and comprehensive pathological analysis. Since the tumor cells in renal cell carcinoma and RH share some morphological features and both express markers such as CD10 and PAX8, some of these renal tumors are difficult to classify.

When diagnosing a renal cell carcinoma, a strict follow‐up plan over years is necessary. Depending on the risk factors; based on pathological stage, comorbidities, and relapse location; post‐treatment surveillance has to be carried out. In intermediate‐ and high‐risk tumors, the European Association of Urology‐panel recommends computed tomography examination of chest and abdomen once a year for 3 years followed by computed tomography once every 2 years. And even in low‐risk tumors, a computed tomography should be performed at least every 2 years.^27^ The mean effective dose for whole body CT is about 14 mSv, which means a significant radiation exposure over the time of follow‐up for each patient. Calandrino and co‐workers evaluated an absolute additional risk of developing second cancer due to CT‐scans of follow‐up protocols between 0, 1%, and 10%. Major risk determinants were tumor pathology and age at exposure.^28^ However, a totally excised benign tumor needs no more surveillance and further radiation exposure can be avoided.

Although renal cell carcinoma is much more common, where there is any doubt, we recommend immunohistochemical stainings for pan‐cytokeratin, S100, NSE, and inhibin‐alpha to avoid the potential for misdiagnosis and to keep in mind a hemangioblastoma as differential diagnosis.

## CONFLICT OF INTEREST

Lukas Oberhammer, Lukas Lusuardi, Thomas Kunit, Martina Hager, Martin Drerup, Daniela Colleselli, Hubert Grießner, and Michael Mitterberger declare that they have no conflicts of interest.

## AUTHOR CONTRIBUTIONS

LO: collected data, wrote the manuscript, and involved in project development. LL, TK, DC, and MD: wrote the manuscript. MH: collected data and wrote the manuscript. HG: collected data. MM: wrote the manuscript and involved in project development.

## ETHICAL APPROVAL

Not applicable.

## CONSENT TO PUBLISH

Written informed consent was obtained from the patient for publication of this Case Report and any accompanying images. A copy of the written consent is available for review by the Editor of this journal.

## Data Availability

Pathological findings were evaluated in the Department of Pathology, Uniklinikum Salzburg, Austria. The CT scan was performed in the Department of Radiology, Uniklinikum Salzburg, Austria. On demand, all the data can be delivered.
